# CircCDK14 Promotes Tumor Progression and Resists Ferroptosis in Glioma by Regulating PDGFRA

**DOI:** 10.7150/ijbs.66114

**Published:** 2022-01-01

**Authors:** Simin Chen, Zhaoyu Zhang, Baoxin Zhang, Qing Huang, Yi Liu, Yi Qiu, Xinmiao Long, Minghua Wu, Zuping Zhang

**Affiliations:** 1 School of Basic Medical Science, Central South University, Changsha 410013, Hunan, China.; 2 Cancer Research Institute, Central South University, Changsha 410013, Hunan, China.; 3 Armed Police Hospital of Hunan Province, Changsha 410013, Hunan, China.; 4 Department of Neurosurgery, Xiangya Hospital, Central South University, Changsha 410013, Hunan, China.; 5 Department of Clinical Laboratory, Yueyang Central Hospital, Yueyang 414000, Hunan, China.

**Keywords:** CircCDK14, Glioma, miR-3938, PDGFRA, Ferroptosis

## Abstract

CircRNAs have garnered significant interest in recent years due to their regulation in human tumorigenesis, yet, the function of most glioma-related circRNAs remains unclear. In this study, using RNA-Seq, we screened differentially regulated circRNAs in glioma, in comparison to non-tumor brain tissue. Loss- and gain-of-function strategies were used to assess the effect of circCDK14 on tumor progression both* in vitro* and *in vivo*. Luciferase reporter, RNA pull-down and fluorescence *in situ* hybridization assays were carried out to validate interactions between circCDK14 and miR-3938 as well as miR-3938 and PDGFRA. Transmission electron microscopic observation of mitochondria, iron and reactive oxygen species assays were employed for the detection of circCDK14 effect on glioma cells' sensitivity to erastin-induced ferroptosis (Fp). Our findings indicated that circCDK14 was overexpressed in glioma tissues and cell lines, and elevated levels of circCDK14 induced poor prognosis of glioma patients. CircCDK14 promotes the migration, invasion and proliferation of glioma cells *in vitro* as well as tumorigenesis *in vivo*. An evaluation of the underlying mechanism revealed that circCDK14 sponged miR-3938 to upregulate oncogenic gene PDGFRA expression. Moreover, we also found that circCDK14 reduced glioma cells' sensitivity to Fp by regulating PDGFRA expression. In conclusion, circCDK14 induces tumor in glioma and increases malignant tumor behavior via the miR-3938/PDGFRA axis. Hence, the miR-3938/PDGFRA axis may be an excellent candidate of anti-glioma therapy.

## Introduction

Glioma is a highly prevalent and aggressive malignant tumor of the central nervous system with poor prognosis. Despite the comprehensive provision of advanced chemotherapy, radiotherapy and surgery, glioma patients experience a poor median survival. Particularly, glioblastoma multiforme (GBM), the most frequent and severe form of the disease, has a median overall survival (OS) of 15 months, that has remained the same for decades 1. In addition to the accelerated proliferation, aggressive invasion and treatment resistance, the severe glioma prognosis also results from limited knowledge of underlying pathways, lack of early diagnosis and efficacious therapy 2. Hence, it is urgent and necessary to examine glioma-related signaling pathways involved in initiation and progression, which may help to explore reliable biomarkers and develop effective treatment strategies.

CircRNAs, a novel type of circular intrinsic noncoding RNAs, are formed via end-splicing and lack the 5' to 3'polarity and a polyadenylated tail 3. Due to this property, circRNAs have high cellular stability because of their tolerance of exonucleases. According to multiple reports, circRNAs express in a tissue specific manner and under pathological conditions 4. Emerging evidences reveal that circRNAs are differentially expressed (DE) in gliomas and are associated with disease progression 5. In glioma, some circRNAs serve as miRNA sponges that sequester miRNAs activity and indirectly regulate target gene expression 6-8, while others behave like RNA binding proteins and regulate protein interaction 9, 10, and yet some others are reported to be translatable 11-13. Overall, much needs to uncover regarding the mechanism of action of circRNAs.

The circRNA-miRNA-mRNA pathway is crucial for glioma development and progression. For example, circHIPK3 accelerated glioma progression via the circHIPK3/miR-654/IGF2BP3 network 14. And overexpressing circ-HIPK3 enhanced proliferative and invasive abilities of glioma cells via sequestering miR-124-3p and raising STAT3 levels 15. In addition, in GBM tissues, circCPA4, circSCAF11, and circ-PITX1 levels are markedly elevated, which results in rapid progression of glioma tumorigenesis via the let7/CPA4, miR421/SP1/VEGFA, miR-379-5p/MAP3K2 pathways, respectively 16-18. Additionally, circPTN stimulated glioma expansion and stemness via sequestering miR-145-5p and miR-330-5p, and increasing Nestin, CD133, SOX9, and SOX2 expression 19. However, due to the function of most circRNAs being unclear, other crucial circRNAs in glioma deserve exploring.

Ferroptosis (Fp) is an iron-based and reactive oxygen species (ROS)-dependent cellular death. It offers a remarkable role in reducing tumorigenesis, carried out via removal of cells deficient in key nutrients or damaged by infection or ambient stress 20. Multiple reports suggest that the oxidative stress pathway is a major stimulator of Fp. Interestingly, despite being under constant oxidative stress (with a fine balance between thiols and catalytic iron), Fp is not common in cancerous environments 21, 22. Several genes, namely, SLC7A11, GPX4, ACSL4, TP53, NRF2 and GLS2 are known to regulate cellular responsiveness to Fp 23-25. Those reports associated Fp to numerous cellular processes like iron homeostasis, redox homeostasis, lipid metabolism, and glutaminolysis. Nevertheless, the roles of circRNAs in Fp are almost unaware.

Herein, we explored the DE circRNAs in glioma by analyzing the levels of circRNAs in glioma tissue and non-tumor brain tissue by RNA-sequencing. We identified circCDK14 as highly significant due to its marked upregulation in glioma tissue and cell lines. Elevated circCDK14 levels were strongly correlated with poor OS. Upon further evaluation, we demonstrated that circCDK14 accelerated glioma progression and inhibited glioma cells' sensitivity to Fp via regulating the miR-3938/PDGFRA axis.

## Materials and Methods

### Clinical samples

Overall, 76 primary glioma samples were collected from the brain tumor patients and 17 non-tumor tissues were excised from the brain trauma or epileptic patients. These samples were from the Department of Neurosurgery, Xiangya Hospital, Central South University (Changsha, China). To preserve freshness, all samples were snap-frozen upon harvest and maintained in liquid nitrogen until processing. We received written informed consent from all participants or guardians of participants and obtained ethical approval from the School of Basic Medical Science, Central South University (Changsha, China).

### RNA sequencing and bioinformatic analyses

RNA sequencing was performed on 5 glioma samples and 4 non-tumor brain tissues by LC Sciences (Hangzhou, China). Total RNA was extracted with the Trizol reagent (Invitrogen, USA), following kit operational guidelines. RNA quality was evaluated with Agilent 2100 with RIN number >7.0. Next, ~5 ug of total RNA was employed for the depletion of ribosomal RNA (rRNA), following the Ribo-Zero™ rRNA Removal Kit (Illumina, San Diego, USA) guidelines. Following rRNA removal, a cDNA library was generated with the VAHTS Total RNA-seq (H/M/R) Library Kit from Illumina, following kit guidelines. The final cDNA library had an average insert size of 300 bp (±50 bp). Finally, the libraries underwent paired-end sequencing with pair end 150 bp reading length, using Illumina Hiseq 4000 (LC Bio, China), based on operational direcctions.

The FastQC software (v0.11.2) assessed fastq formatted raw data sequencing quality. Data with poor quality were eliminated with the FASTQC software (v2.3.2). The remaining high-quality data were subsequently aligned to the human reference genome (GRCh38/hg38) using the Hisat2 software (v2.0.13) with default parameters. Sequencing data that failed to align to reference genome were entered into circRNA analysis via determination of the back splicing event using Find_circ (v1.0) and CIRCexplorer2. Perl staistics package of software (v3.32.10) was employed for the analysis of the differentially regulated circRNAs.

### Cell culture

The normal brain glial cell line HEB was acquired from the Cell Bank of the Type Culture Collection of the Chinese Academy of Sciences (Shanghai, China). Human glioma SF126 cells were acquired from the Cell Research Institute of Peking Union Medical College (Peking, China). Human glioblastoma-derived cell lines U251 and U87 were retrieved from the American Type Culture Collection (ATCC, USA). U87, U251, and HEB cells were maintained in DMEM (Hyclone, USA) medium with high glucose, and SF126 cells were cultured in MEM (Hyclone, USA) medium, containing 10% fetal bovine (Biosharp, China) and 1% penicillin/streptomycin (Solarbio, China) serum at 37 °C in a 5% CO_2_ incubator.

### Quantitative real-time PCR

Total RNA isolation was done with the TRIzol reagent (Invitrogen, USA). After quality control and quantification, individual RNA samples (1μg each) were converted to cDNA with the Prime Script RT reagent Kit (Takara, Japan), and detected by Hieff® qPCR SYBR Green Master Mix (Yeasen, China) on ABI Prism 7500 sequence detection system (Applied Biosystems, USA). The nuclear and cytoplasmic fractions were isolated with a BestBio Nuclear and Cytoplasmic Extraction Reagents Kit (BestBio, China). GAPDH and small nuclear U6 were employed as endogenous controls. qRT‐PCR was conducted using primers obtained from Tsingke (Changsha, China). The primer sequences used are described in Table S1.

### Nucleic acid electrophoresis

In tris-acetic acid-EDTA (TAE) buffer, cDNA and gDNA amplification products were separated on 2% agarose gel and added to the DNA Marker (100-600bp) (Sango Biotech, China) to isolate DNA for 20 minutes via 120 V electrophoresis. Use ultraviolet light to detect the band.

### RNase R assay

The original RNA (2μg) was added to 2U RNase R (GeenSeed, China) to digest a large amount of linear RNA at 37 °C for 30 minutes. Then RNase R was inactivated at 70 °C for 10 minutes and reverse transcription reaction was followed. The internal control (GAPDH) in the other untreated group is used as the calculation standard, and then circCDK14 and CDK14 levels are assessed by qRT-PCR.

### RNA Fluorescence *in situ* hybridization (FISH)

FISH was conducted with the help of targeted probes for the back-splice region of circCDK14 and miR-3938 sequences. Cy3-labeled circCDK14 probes and FAM-labeled miR-3938 probes were synthesized by Servicebio (Wuhan, China). Nuclei staining were done with DAPI. Finally, probe signals were measured with the Fluorescent *in situ* Hybridization Kit (RiboBio, China) following its directions and images were captured with a Zeiss LSM710 laser scanning confocal microscope (Zeiss Instruments, Germany).

### Cell proliferation

The proliferation of cells was determined in three separate experiments quadruplicate by Cell Counting Kit-8 (Yeasen, China). Briefly, individual cell types (2000 cells/well) were cultured in 96-well plates for 96 h and fresh medium was replaced every day. During the last hour of culture, 10 µL of CCK8 solution were added for each well and absorbance read at 450 nm in a microreader.

### Wound healing and transwell invasion assays

In the wound healing assay, individual cell types (1×10^6^ cells/well) were grown to confluent monolayers on 6-well plates, and the cells were scratched with a 10 ul pipette tip. The migration distance was calculated using the equation: migration distance=S _time-zero_-S _time-point_. The assays were performed in triplicates.

In the transwell invasion assay, individual types of cells (1×10^4^/well) were grown in FBS-free medium in the top chambers of 24-well migration plates. Medium containing 10% FBS was introduced to the bottom chambers to provide chemoattractant factors. After 24 h in an incubator, cells on the top chamber were wiped away. Those on the bottom were fixed with 4% paraformaldehyde and stained with 0.1% crystal violet. Cell counting was done under a microscope at 100× magnification in a blinded manner. Three independent experiments were conducted in triplicates.

### Cell transfection

The short interfering RNA (siRNA) for knocking out hsa_circ_0001721 (circCDK14) or PDGFRA, miRNA mimics (miR-29b-1-5p, miR-185-3p, miR-519d-3p, miR-3938) and miR-3938 inhibitor were acquired from RiboBio (Guangzhou, China) and siRNA-NC (negative control), NC for mimics, and NC for inhibitor used as a control. The overexpression plasmids (pcDNA3.1-circRNA Mini vector) of circCDK14 were acquired from Genscript (Nanjing, China). U87 and U251 cells were plated in 6-well plates for 24 h to achieve 70-80% confluence. And 12 μl Lipofectamine 2000 (Invitrogen, USA) was used to transfect siRNA or siRNA-NC with a final concentration of 50 μM into U251 cells. Similarly, 2000 ng circCDK14 overexpression plasmid or empty plasmids were also transfected into U87 cells. The transfected cells were incubated at 37 °C and 5% CO_2_ for 24-36h. The siRNA sequences for circCDK14 are listed below: si-circRNA1: CAGCTCGATATGTGTCACA; si-circRNA2: CTCGATATGTGTCACAAAG; miR-3938 inhibitor: CCGGGUUAUCUACAAGGGAAUU; siPDGFRA: GCATCACAATGCTGGAAG.

### Animal studies

4-week-old female BALB/c nude mice were acquired from the National Laboratory Animal Center (Shanghai, China). Overall, 10 mice (n=5 each group) were implanted with U251 cells stably knockdown circCDK14 by lentiviruses carrying sh-circCDK14 (Wanleibio, China), or control U251 cells with lentiviruses carrying sh-NC (Wanleibio, China). 5 × 10^6^ cells were resuspended in phosphate‐buffered saline (100 µl) and Matrigel substrate (100 µl) and injected into the right flank of nude mice. Tumor volume was documented once 7 days after implantation. Tumor volume was calculated using calipers and the formula (V mm^3)^ = length× width^2^/2. The animals were sacrificed at 35 days after injection and the tumors were isolated and weighed immediately. The Animal Research Committee of Xiangya School of Medicine, Central South University approved all the study experiments.

### miRNA binding site prediction and Luciferase reporter assay

The miRNA bindig sites were predicted using several bioinformatics databases miRanda (http://www.microrna.org), TargetScan (http://www.targetscan.org/), RNA-hybrid (https://bibiserv.cebitec.uni-bielefeld.de/rnahybrid/), circBank (http://www.circ-bank.cn) and starBase (http://starbase.sysu.edu.cn/index.php). Dual‐luciferase reporter (DLR) plasmids were constructed using pmirGLO dual‐luciferase vectors (Promega, USA), cloned circCDK14-WT, circCDK14-MUT, PDGFRA -WT and PDGFRA-MUT into the vector, respectively. All luciferase reporter vectors were synthesized by Gen-Script (Nanjing, China). U87 and U251 cells were plated into 96-well plates and were co-incorporated 50 μM miRNA mimics or inhibitor with 100 ng pmirGLO dual‐luciferase vectors and 1 μl Lipofectamine 2000. After 48 h, luciferase activity was assessed using a DLR Kit (Promega, USA).

### RNA pull-down assay

The biotinylated circCDK14 and PDGFRA probes were purchased from RiboBio (Guangzhou, China). Briefly, 4×10^7^ U87 and U251 cells were harvested and fixed with 1% formaldehyde. Then added 1.7 ml lysis buffer and the cells were lysed on ice for 10 minutes. CircCDK14 or PDDGFRA probes and the corresponding NC probes were added to different samples, hybridized at 37 °C for 30 min, denatured at 50 °C for 5 min, and hybridized at 37 °C for 90~180 min. Then C-1 magnetic beads (Life Technologies) were incubated for 30 minutes and collected. This was followed by a rinse wash buffer, before elution of RNA bound complexes and extraction of RNA using a RNeasy Mini Kit (QIAGEN) for qRT-PCR.

### Bioinformatic analyses of PDGFRA

Using the CGGA database (http://www.cgga.org.cn.), we collected the mRNA expression and clinical data 26. Gene expression data from TCGA cancer dataset and GTEX dataset were downloaded and normalized to compare the expression between cancer and normal samples.

We followed the method of Liu Z et al to calculate the index that represents the degree of Fp, using gene expression data for core Fp positive and negative regulators 27. The gene set enrichment score (ES) of genes that modulated Fp was calculated with the single sample gene set enrichment analysis (ssGSEA) in the R package 'GSVA' 28, and the normalized differences between the ES of the pro-regulators minus the anti-regulators was described as the Fp potential index (FPI) 27.

### Western blot

Cell lysis was done in chilled RIPA lysate buffer (Beyotime, China) with protease inhibitor and phosphatase inhibitor. Equal amounts of total protein were separated on SDS-PAGE gels at 100 V for 1.5 h, before transfer to 0.45 μm PVDF membrane (NEST, China). Blocking was done in 5% non-fat dry milk in TBST with subsequent overnight incubation with primary antibodies at 4 °C. Primary antibodies were against PDGFRA (1:1000, abcam), p-PDGFRα (1:1000, abcam),β-tubulin (1:1000, wanleibio, China), GAPDH (1:5000, abcam), vimentin (1:500, wanleibio, China), E-cadherin (1:500, wanleibio, China), ZEB1 (1:500, wanleibio, China), GPX4 (1:1000, abcam), NRF2 (1:1000, abcam) and SLC7A11 (1:1000, abcam). Upon three TBST washes, membranes were incubated with secondary antibody, before visualization with enhanced chemiluminescence (ECL) reagent (Yeasen, Shanghai, China).

### Iron assay

The iron assay kit (Leagene, China) adopts spectrophotometric method to detect iron with ferrozine as the substrate. Following the guidelines, 4×10^3^ cells/well was plated in 96-well plates. After circCDK14 interference fragments was transfected into U251 cells, cells were treated with erastin (10 uM) (Selleck, USA) for 48 hours. Then, the sample was incubated with 200 ul Fe^2+^ assay buffer and 75 ul standard solution for 15 min at 37 °C. And absorbance was read with a microplate reader at 562 nm. Absorbance values were calibrated to a standard concentration curve to calculate the concentration of iron.

### Reactive oxygen species (ROS) assay

The Reactive Oxygen species assay kit (Biosharp, China) is used for reactive oxygen detection by H2DCFH-DA fluorescent probe. 5×10^5^ cells/well was grown in 6-well plates and exposed to H2DCFH-DA (10 µM) and incubated in a 37 °C cell incubator for 30 min. After PBS wash, fluorescence strength was observed by a fluorescence microscope (Leica, Germany). And ImageJ is used to measure fluorescence strength.

### Transmission electron microscopy

Cells were fixed with 1ml fixative solution (2.5% glutaraldehyde) and placed at 4 °C for 4-6 hours. Ultrathin sections (65 nm) were made and stained with 1% uranyl acetate and 0.1% lead citrate, before examining under a transmission electron microscope (JEOL, Pleasanton, California, USA).

### Statistical analysis

Data analysis was done with Student's unpaired t-test and one-way ANOVA in the GraphPad Prism 8 software. All experiments were repeated at least 3 times. Pearson's correlation coefficient was employed for correlation examination. Data presented as mean ± standard deviation (SD). A *P* value less than 0.05 was set as significance threshold.

## Results

### Identification of circCDK14 in glioma via RNA-Seq analysis

**Figure 1:**
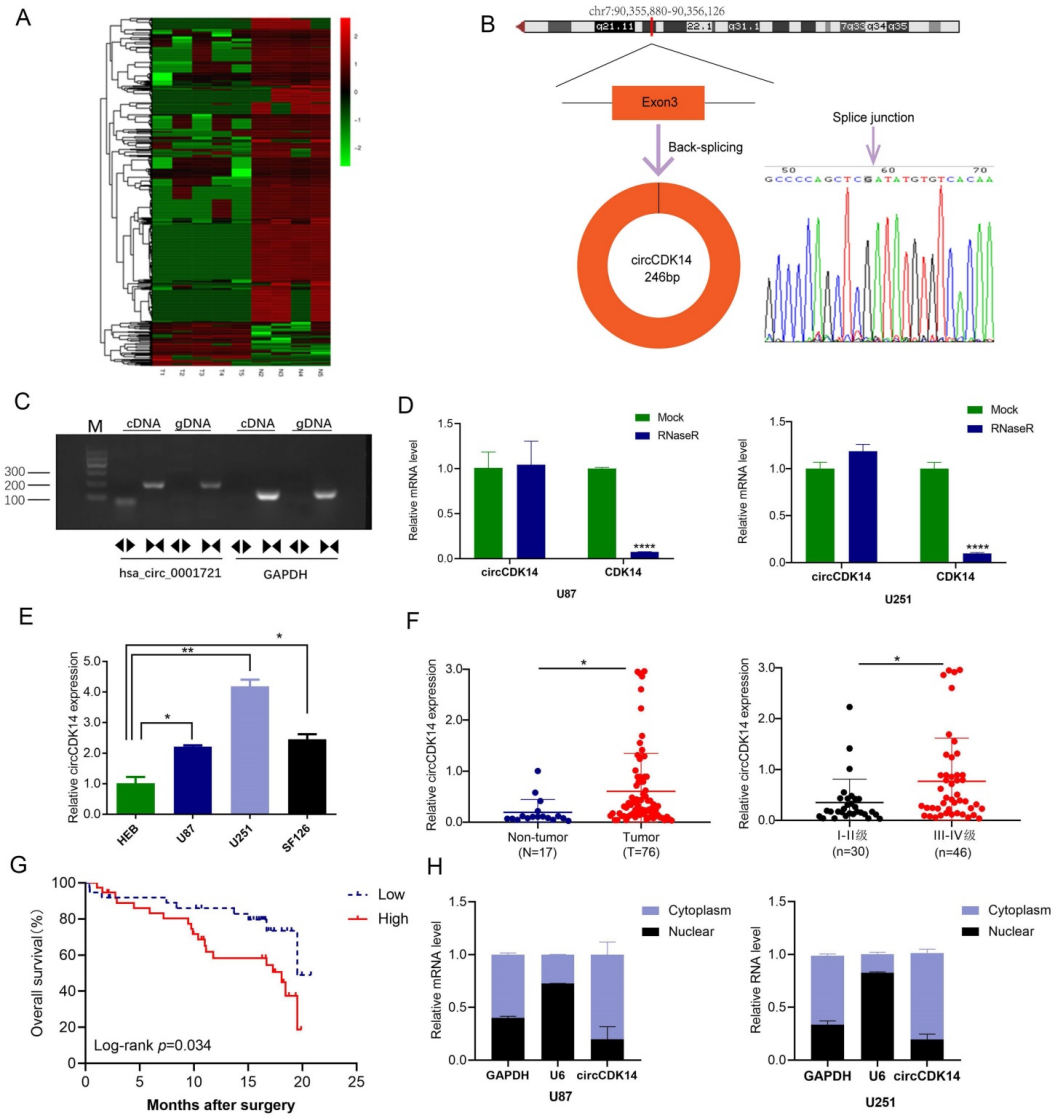
** Expression and characterization of circCDK14 in human glioma. (A)** Clustered heatmap of significant differentially regulated circRNAs in glioma tissues and non-tumor brain tissues (|fold change >2, *p*<0.05). Red, up- and green, down-regulated. **(B)** Schematic illustration of genomic location and formation of circCDK14 (hsa_circ_0001721), derived from exons 3 circularization of CDK14 gene. The back-splice junction sequences of circCDK14 were confirmed by Sanger sequencing. Arrow, “head-to-tail” splice junction site. **(C)** PCR was analyzed to the circular RNA characterization of the circCDK14 (hsa_circ_0001721), using the divergent and convergent primers amplifying from the cDNA and gDNA of U251 cells, respectively. **(D)** qRT-PCR analysis of circCDK14 and linear CDK14 mRNA, in presence or absence of RNase R treatment for 30 min. **(E)** CircCDK14 levels, as quantified by qRT-PCR in human glioma cell lines (U87, U251 and SF126) and normal brain glial cells (HEB). circCDK14 expression level were significantly elevated in glioma cells, relative to HEB cells. **(F)** CircCDK14 levels in 76 glioma and 17 non-tumor brain tissues, and in 30 patients with I-II grade glioma and 46 patients with III-IV grade glioma. **(G)** Overall survival curve, based on circCDK14 levels, plotted with Kaplan-Meier methods and analyzed by rank test. **(H)** qRT-PCR analysis of circCDK14 expression location using nuclear and cytoplasmic fractions of U87 and U251 cells. U6 small nuclear RNA and GAPDH was endogenous control. Data expressed as mean±SD. *p< 0.05; **p< 0.01.

Overall, 42,994 circRNAs were identified by RNA-Seq in 5 glioma tissues and 4 non-tumor brain tissues. After fold-change filtering (|log2(fold change)| > 1) and Student's *t*-testing (*p* value < 0.05) of the DE circRNAs data, 1248 DE circRNAs were recognized. Relative to non-tumor brain tissues, glioma tissues exhibited 158 highly expressed and 1090 lowly expressed circRNAs. Volcano plots provided a visual display of the DE circRNAs in glioma tissues (Figure S1). Additionally, using hierarchical clustering, we demonstrated that the circRNA expression patterns were remarkably different between the glioma and control groups (Figure 1A). To confirm the results of RNA-Seq, seven down-regulated and five up-regulated circRNAs were detected by qRT-PCR in 9 sequencing samples. The results were consistent with that of sequencing (Figure S2A and S2B), indicating that results of RNA-Seq are reliable.

Among the circRNAs whose expression had been validated in glioma, circCDK14 (hsa_circ_0001721), derived from exons 3 of CDK14 (cyclin-dependent kinase 14), is interested by us. Because its parental genes CDK14 is a cell cycle-dependent kinase, which is involved in the occurrence and development of tumor, including glioma 29, 30. However, the function of circRNAs derived from CDK14 in glioma remains unknown. The length of circCDK14 is 246bp and is locates in chr.7:90,355,880-90,356,126 (Figure 1B). Using PCR analysis, we revealed that divergent primers were successful in producing circCDK14 from cDNA, but not genomic DNA (gDNA). Conversely, convergent primers amplified the linear CDK14 from both c-and gDNA (Figure 1C). Moreover, with qRT-PCR, we revealed that circCDK14 can resist RNase R, while CDK14 mRNA was destroyed by RNase R in U87 and U251 cells (Figure 1D). Lastly, Sanger sequencing of the PCR products using divergent primers further validated the existence of a splice junction in circCDK14 (Figure 1B). These data indicated that circCDK14 had the characteristics of circular RNA.

To further examine circCDK14 levels in glioma, we employed qRT-PCR to assess circCDK14 levels in HEB, U251, U87 and SF126 cells, 76 glioma and 17 non-tumor brain tissues. Based on our data, circCDK14 levels in U251, U87 and SF126 cells were markedly higher than that of normal brain glial cells (HEB) (Figure 1E). And the circCDK14 levels were markedly elevated in glioma tissues, relative to non-tumor brain tissues. Moreover, expression level of circCDK14 in III-IV grade glioma was higher, compared to I-II grade glioma (*P* < 0.05, Figure 1F). To explore the clinical significance of circCDK14 in glioma, the median expression level of glioma tissues was selected as the threshold for separating the dataset into high or low circRNAs levels. Kaplan-Meier analysis demonstrated an inverse relationship between circCDK14 levels and OS time of glioma patients (*P* < 0.05, Figure 1G). Additionally, we assessed localization of circCDK14 in U87 and U251 cells. The results show that circCDK14 is mainly present in the cytoplasm (Figure 1H).

### circCDK14 accelerates proliferation, migration and invasion of glioma

**Figure 2:**
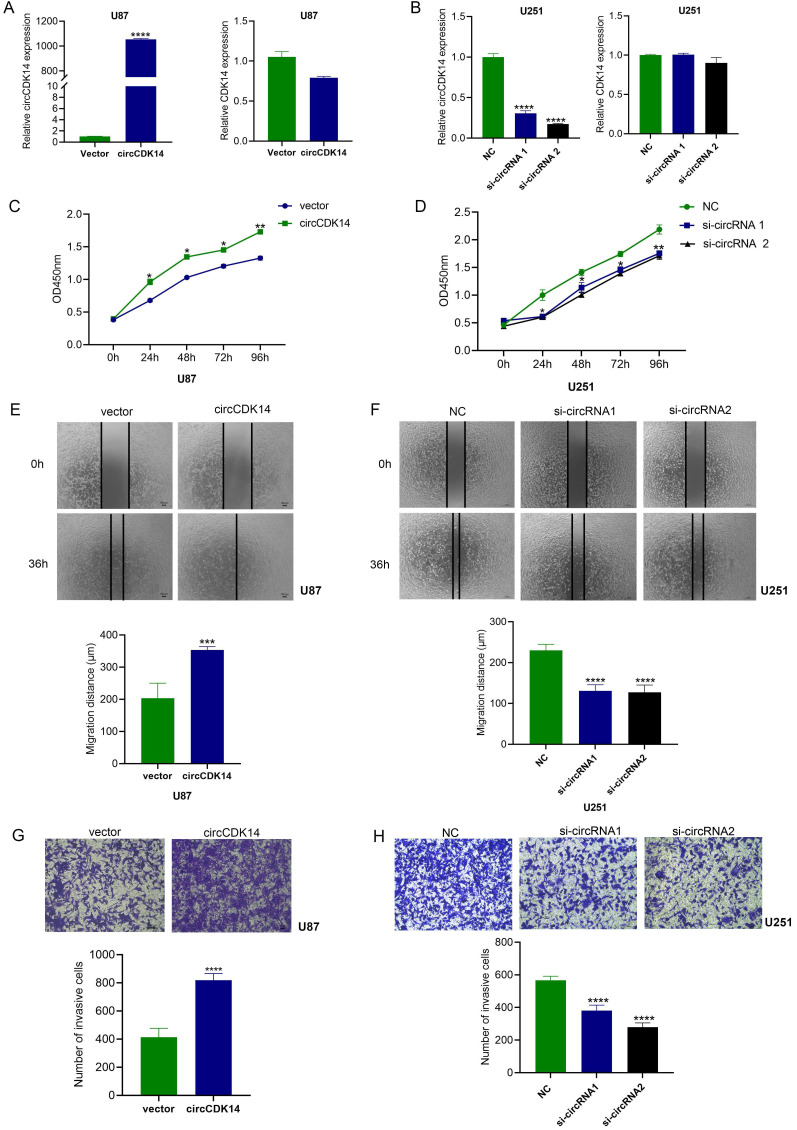
** circCDK14 promotes the proliferation, migration and invasion of glioma. (A and B)** Levels of circCDK14 and host gene CDK14 mRNA in U87 cells and U251 cells after transfection of circCDK14 overexpression plasmid or circCDK14 interference sequences, were detected by qRT-PCR. **(C)** Overexpressing circCDK14 promotes U87 cell proliferation, as determined by CCK-8. **(D)** CircCDK14 knockdown inhibits U251 cell proliferation, as determined by CCK-8. **(E and F)** Wound healing assay determined the migrating capacity of glioma cells after various treatments. **(G and H)** Transwell assay was used to detect the invasion activity of glioma cells following different treatments. Both scale bars are 100 µm. All experiments repeated thrice and averaged (mean±SD). Unpaired Students's t-test was employed for all data analyses. *p< 0.05; **p< 0.01; ***p< 0.001; ****p< 0.0001.

To evaluate the bioactivity of circCDK14 in glioma, the circCDK14 sequence was cloned into pcDNA3.1-circRNA Mini vector that consisted of two cyclization elements on the back-splicing site. Two siRNAs that specifically targeted the back-splicing site were also designed for a loss-of-function study. Based on the expression level of circCDK14 in glioma cells (Figure 1E), U87 cells were used to overexpress circCDK14 and U251 were used to knockdown. The results showed that increased circCDK14 expression in U87 cells markedly elevated after ectopic expression. CircCDK14 silencing with siRNA1 and siRNA2 in U251 cells resulted in a significant decrease in circCDK14 levels. The endogenous linear CDK14 mRNA levels, however, did not alter with circCDK14 overexpression or silencing of in glioma cells (Figure 2A and 2B). Overexpressing circCDK14 markedly increased U87 cell proliferation (*P* < 0.001, Figure 2C), whereas, circCDK14 silencing drastically suppressed U251 cell proliferation (*P* < 0.001, Figure 2D). Moreover, overexpressing circCDK14 all remarkably accelerated both migration and invasion of U87 cells (*P* < 0.001, Figure 2E and 2G), whereas, circCDK14 knockdown drastically reduced both activities in U251 cells (*P* < 0.001, Figure 2F and 2H).

### CircCDK14 sequesters miR-3938 activity in glioma

**Figure 3:**
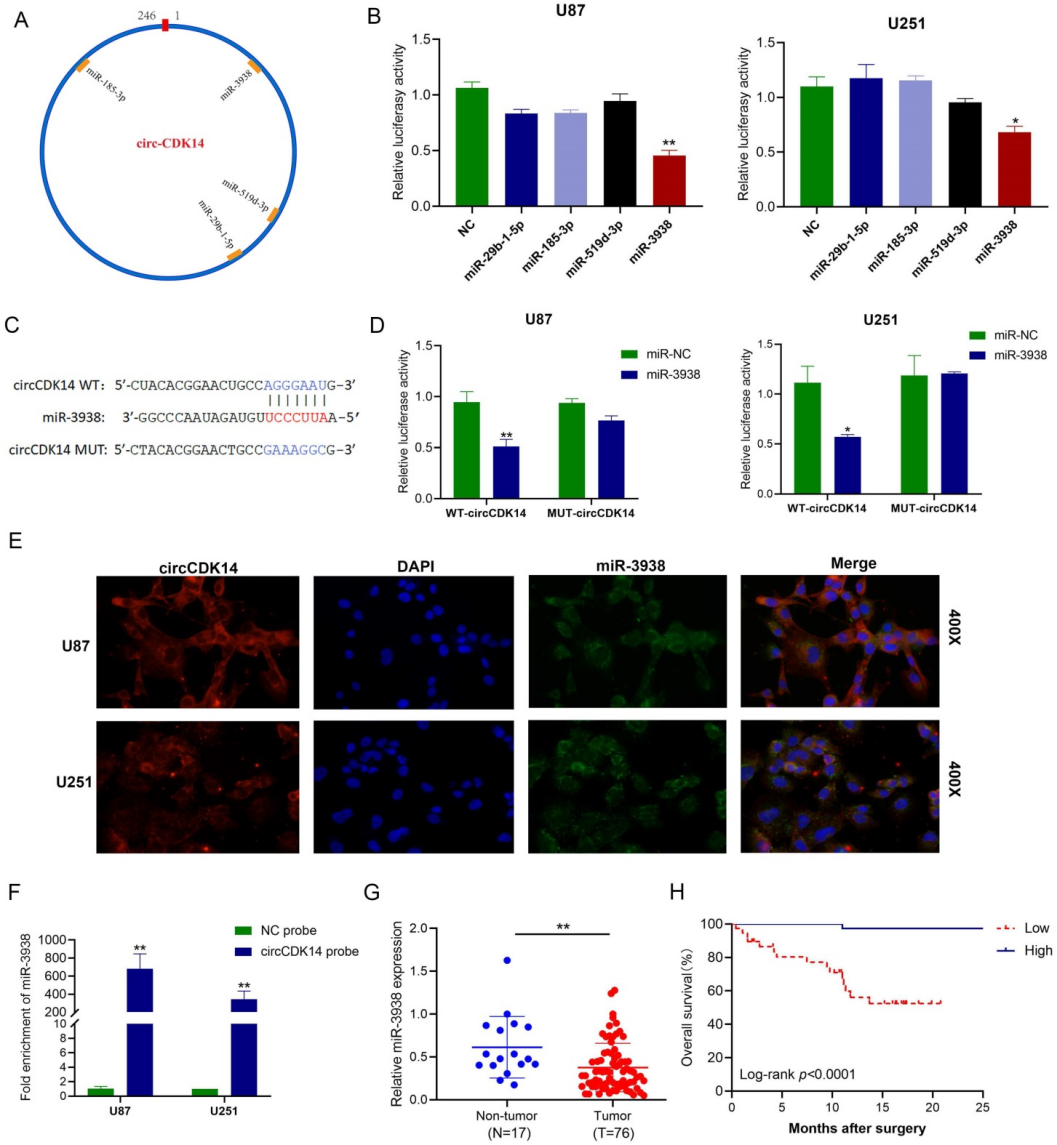
** circCDK14 acts as sponge for miR-3938 in glioma. (A)** Illustrations of the estimated docking sites of miRNA candidates to circCDK14. **(B)** Luciferase activity of pmirGLO-circCDK14-WT in cells after co-incorporation with miRNAs mimics. **(C)** Estimated docking sites of miR-3938 with circCDK14 was predicted. circCDK14 WT, circCDK14 wild-type luciferase reporter; circCDK14 MUT, circCDK14 mutantluciferase reporter. **(D)** U251 and U87 cells were co-transfected with pmirGLO -circCDK14-WT or pmirGLO -circCDK14-MUT and miR‐3938 mimics or negative control. Luciferase activity measured with luciferase reporter assays. **(E)** Colocalization between circCDK14 and miR-3938 were observed by RNA *in situ* hybridization (FISH) in U87 and U251 cells. DAPI staining was used for the nuclei. CircCDK14 probe was Cy3-labeled, and miR-3938 probe was FAM-labeled. **(F)** RNA pull-down assay was conducted to show the relative miR-3938 level in U87 and U251 cells lysates pulled down by circCDK14. **(G)** The expression of miR-3938 in 76 glioma and 17 non-tumor brain tissues were detected by qRT-PCR. **(H)** The overall survival curve, based on miR-3938 levels, was plotted with Kaplan-Meier methods and analyzed by rank test. All experiments repeated three times and data averaged and expressed as mean±SD.*p< 0.05; **p< 0.01.

Given that circCDK14 was largely localized in the cytoplasm, we speculated that circCDK14 may sequester miRNA in glioma. To determine its target miRNAs, miRNAs putative binding sites of circCDK14 were predicted by four databases (circBank, starBase, miRanda and RNAhybrid). Four miRNAs (miR-29b-1-5p, miR-185-3p, miR-519d-3p and miR-3938) were selected from the region that overlapped with four databases (Figure 3A). Next, we conducted luciferase reporter assay with pmirGLO-WT-circCDK14 and miRNA mimics co-transfecting into U87 and U251 cells. The results showed that only miR-3938 mimics significantly reduced the relative luciferase activity (Figure 3B). In order to further confirm the results, pmirGLO-MUT-circCDK14 reporter was also constructed according to potential binding site (Figure 3C). However, miR-3938 mimics couldn't reduce the luciferase activity of pmirGLO-MUT-circCDK14 reporter in U87 and U251 cells (Figure 3D), indicating that miR-3938 can directly bind to circCDK14. As expected, RNA FISH demonstrated that miR-3938 and circCDK14 interacted with each other in the cytoplasm of U251 and U87 cells (Figure 3E). We further validated this interaction with RNA pull-down assay with biotinylated circCDK14 probe (Figure 3F), indicating that miR-3938 can be sponged by circCDK14.

The expression of miR-3938 in glioma tissue and non-tumor brain tissue was detected by qRT-PCR. We demonstrated that miR-3938 levels were drastically downregulated in the glioma tissues, relative to controls (Figure 3G). Moreover, the expression level of miR-3938 in I-II grade glioma was higher than that in III-IV grade glioma (*P* < 0.05, Table S2). Additionally, Kaplan-Meier analysis showed positive relationship between miR-3938 levels and OS time of glioma patients (*P* < 0.001, Figure 3H). The inhibition of miR-3938 expression could counteract the abilities of proliferation, migration and invasion in circCDK14 knockdown U251 cells (Figure S3A-S3E). These data suggested that miR-3938 could not only be sponged by circCDK14 but also reverse its biological function in glioma.

### CircCDK14 regulates PDGFRA expression via miR-3938

**Figure 4:**
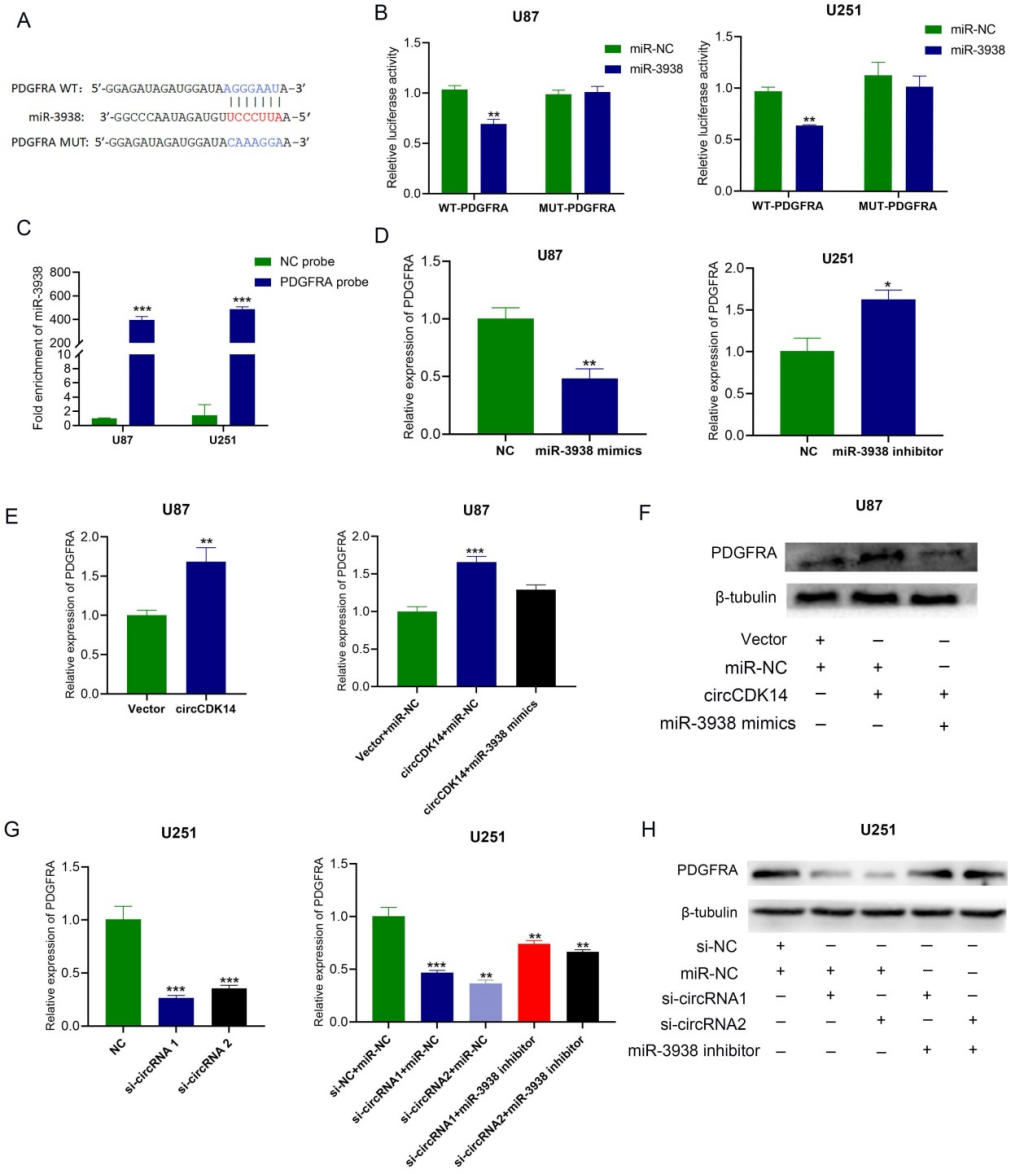
** circCDK14 regulates PDGFRA expression via miR-3938. (A)** Putative binding sites of miR-3938 with PDGFRA was predicted. PDGFRA WT, PDGFRA wild-type pmirGLO luciferase reporter; PDGFRA MUT, PDGFRA mutant pmirGLO luciferase reporter. Wild-type and mutated 3'-UTR sequences of PDGFRA are shown. **(B)** U251 and U87 cells were co-transfected with pmirGLO-PDGFRA -WT or pmirGLO -PDGFRA -MUT and miR‐3938 mimics or negative control. Luciferase activity was measured with luciferase reporter assays. **(C)** RNA pull-down assay was conducted to show the relative miR-3938 level in U87 and U251 cells lysates pulled down by PDGFRA **(D)** PDGFRA expressed was affected by miR-3938 levels. PDGFRA mRNA levels, as evidenced by qRT-PCR in U87 cells incorporated with miR-3938 mimics and U251 cells incorporated with miR-3938 inhibitors. **(E and F)** U87 cells were incorporated with circCDK14 plasmid or co-transfected with circCDK14 plasmid and miR-3938 mimics. RT-PCR and Western blot detected PDGFRA mRNA and protein expressions **(G and H)** U251 cells were incorporated with circCDK14 interference sequences or co-transfected with circCDK14 interference sequences and miR-3938 inhibitors. RT-PCR and Western blot detected PDGFRA mRNA and protein levels. All experiments repeated three times and data averaged and expressed as mean±SD. *p< 0.05, **p< 0.01, ***p< 0.001.

To identify the downstream target gene of miRNA-3938, three databases (namely, TargetScan, miRanda and RNAhybrid) were employed to estimate and PDGFRA (Platelet derived growth factor receptor alpha) was found to have potential binding site with miR-3938. The expression of miR-3938 and PDGFRA in HEB, U87, U251 and SF126 cells were detected by qRT-PCR. The results showed that a negative correlation tendency was found between the expression of miR-3938 and PDGFRA (Figure S4A and S4B). According to the binding site between miR-3938 and PDGFRA, PDGFRA-WT and PDGFRA-MUT luciferase reporters were constructed (Figure 4A). The results of luciferase reporter assay showed that miRNA-3938 mimics significantly reduced the luciferase activity of PDGFRA-WT reporter while that of PDGFRA-MUT reporter didn't change compared with the control in U251 and U87 cells (Figure 4B). The results of RNA pull-down assay with biotinylated PDGFAR probe also indicated the binding of miRNA-3938 and PDGFRA (Figure 4C). The results of qRT-PCR showed miRNA-3938 mimics markedly reduced PDGFRA mRNA levels in U87 cells, whereas, miRNA-3938 inhibitors increased the mRNA expression of PDGFRA in U251 cells (Figure 4D). All the above results revealed that PDGFAR was the direct target gene of miR-3938.

The previous results indicated that circCDK14 acted as sponge of miR-3938, indicating circCDK14 may regulate PDGFRA expression. Then, qRT-PCR and Western blot were employed for the detection of mRNA and protein expression of PDGFRA while overexpression or knockdown of circCDK14 in glioma cells. PDGFRA mRNA and protein levels in U87 cells were markedly increased after circCDK14 overexpression and PDGFAR mRNA and protein levels in U251 cells were significantly decreased after circCDK14 knockdown (Figure 4E and 4F). Moreover, Increasing PDGFAR mRNA and protein expression in U87 cells after circCDK14 overexpression could be counteracted by miR-3938 upregulation. And decreasing PDGFAR mRNA and protein expression in U251 cells could be compensated by miR-3938 inhibitors (Figure 4G and 4H). Together, our results show that circCDK14 regulates PDGFAR expression via sponging miR-3938.

### Downregulated PDGFRA turnovers circCDK14-driven malignancy

**Figure 5:**
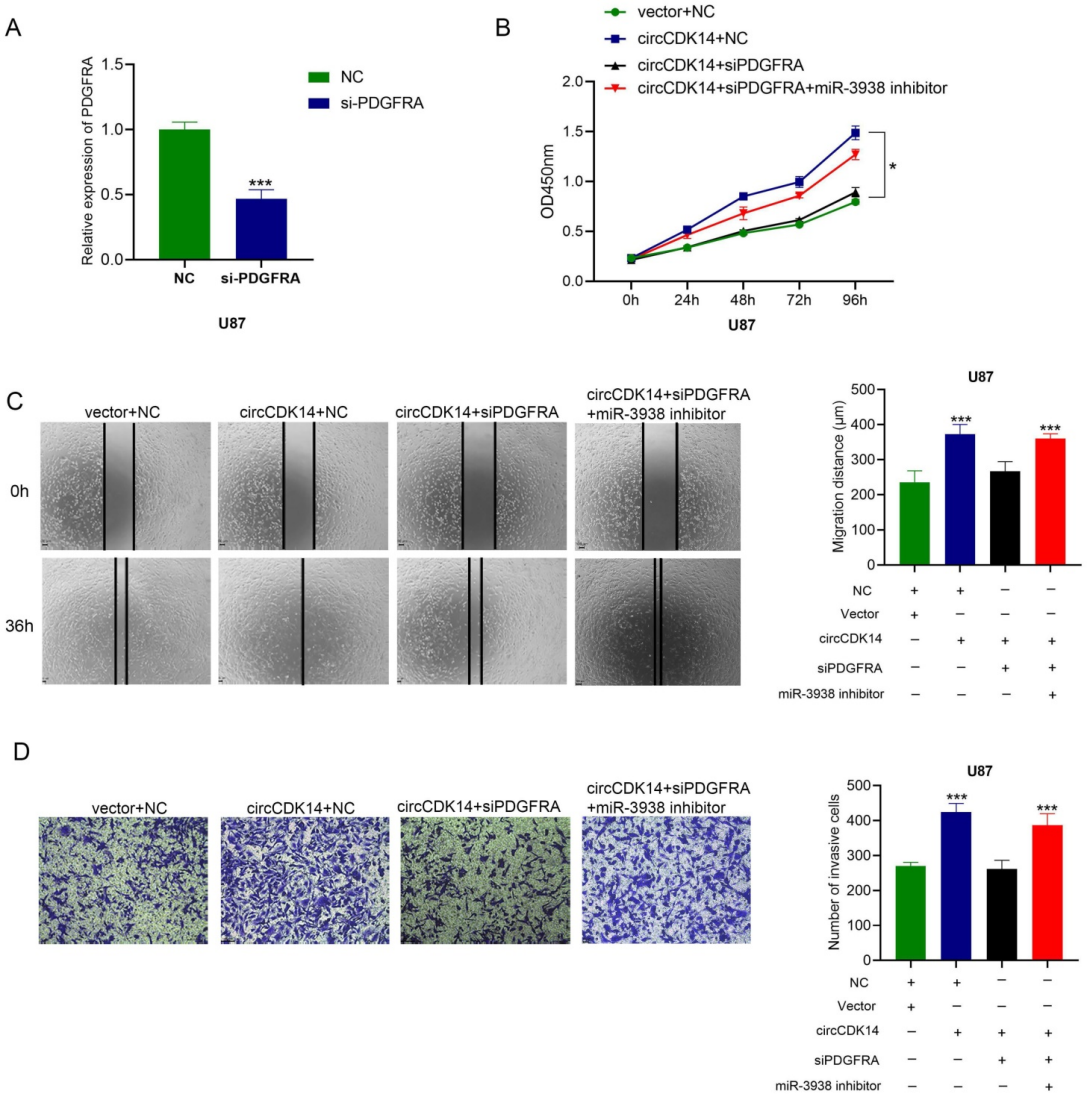
** Downregulated PDGFRA turnovers circCDK14 induced malignant phenotype. (A)** qRT-PCR detects the mRNA PDGFRA levels after transfection of si-PDGFRA. **(B)** Cell proliferation was measured by CCK8 in U87 cells following different treatments. **(C)** Wound healing assay detected U87 cell migrating capacity after various treatments. **(D)** Transwell assay detected the invasion activity of U87 cells following various treatments. All experiments repeated three times and data averaged and expressed as mean±SD. *p< 0.05, ***p< 0.001.

To further examine whether circCDK14-driven glioma progression occurs via sponging miR-3938, rescue experiments were performed. Overexpression circCDK14 U87 cells were treated with siPDGFRA or siPDGFRA and miR-3938 inhibitors. Then CCK8, wound healing and transwell invasion assays were conducted. Overexpressing circCDK14 accelerating proliferation, migration and invasion promotion was abrogated by knockdown of PDGFAR, while miR-3938 inhibitors could reverse the blocking of siPDGFAR (Figure 5A-5D). These results showed that PDGFAR downregulation reverses the circCDK14-induced malignant phenotype.

### PDGFRA expression is upregulated in glioma and negatively associated with Fp

**Figure 6:**
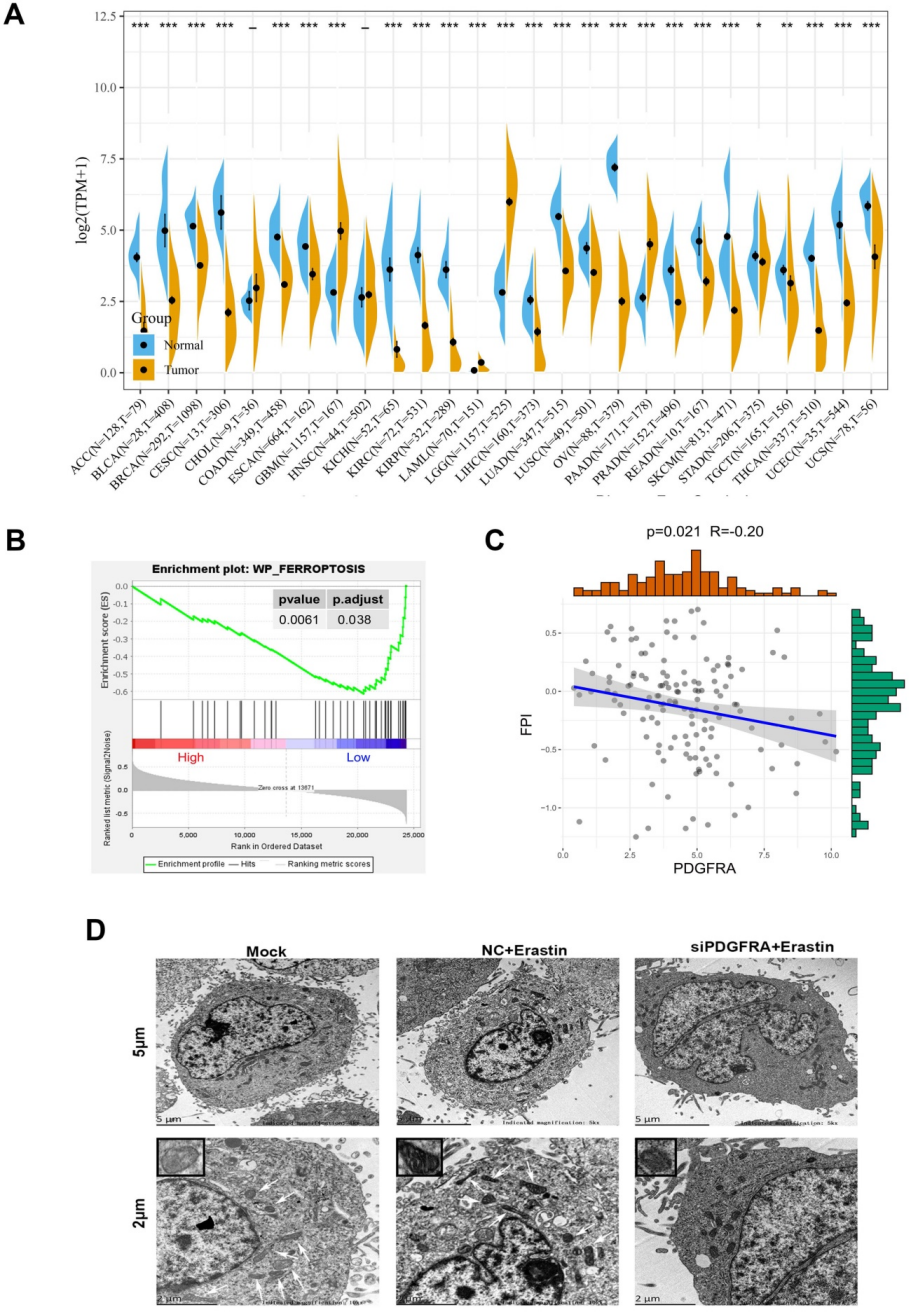
** PDGFRA expression is upregulated in glioma and negatively associated with ferroptosis. (A)** Pan-cancer PDGFRA levels between TCGA-derived tumor tissues and TCGA- and GTEx-derived normal tissues. Asterisks (*P < 0.05, **P < 0.01, ***P < 0.001, independent variable t-test) represents marked alterations in median PDGFRA mRNA levels between tumors and normal control. **(B)** GSEA indicated significant enrichment of Fp phenotype in the low PDGFRA expression patients. **(C)** The Spearman's correlations between PDGFRA expression and the FPI. TCGA, The Cancer Genome Atlas; GTEx, Genotype-Tissue Expression; GSEA, Gene Set Enrichment Analysis; FPI, Fp potential index. **(D)** Transmission electron microscope was used to observe mitochondria in U251 cells with different treatment. Scale bar: 5 µm and 2 µm. In the lower set of figures, arrows point to the mitochondria. The small box in the upper left corner shows the enlarged mitochondria. Mock, control group; NC+Erastin, U251 cells transfected by negative control sequence following 10 µM erastin treatment for 24 h; siPDGFRA +Erastin, U251 cells transfected by PDGFRA interference sequence1 following 10 µM erastin treatment for 24 h.

To know more functions of PDGFRA, the expression differences of PDGFRA were analyzed in 27 types of tumors in the TCGA database and GTEX database compared with normal tissues. The results showed that PDGFRA expression are upregulated only in GBM, LGG (Low grade glioma) and PAAD (Pancreatic adenocarcinoma) (Figure 6A). We further analyzed 139 sequencing data of high-grade glioma from 325 samples in CGGA database. Using functional enrichment analysis via the Gene Set Enrichment Analysis tool, we identified enriched gene sets or pathways within our expression dataset. The results showed that the low expression PDGFRA group was enriched with Fp genes (Figure 6B). In addition, we used the Fp potential index (FPI) model to score the 139 sequencing samples. Based on our data, the PDGFRA levels were inversely associated with FPI score (Figure 6C). To further verify the role of PDGFRA in Fp, mitochondria morphology of glioma cells was observed by transmission electron microscopy. Compared with U251 cells treatment with erastin, contracting mitochondria, reducing cristaes and increasing membrane density, and the changes in mitochondria were more obvious in PDGFRA knockdown U251 cells treatment with erastin. Those results implicated that PDGFRA expression was negatively associated with Fp (Figure 6D). As circCDK14 regulates PDGFRA expression via sponging miR-3938, the roles of circCDK14 in Fp deserve being explored.

### CircCDK14 inhibits glioma cells' sensitivity to Fp

**Figure 7:**
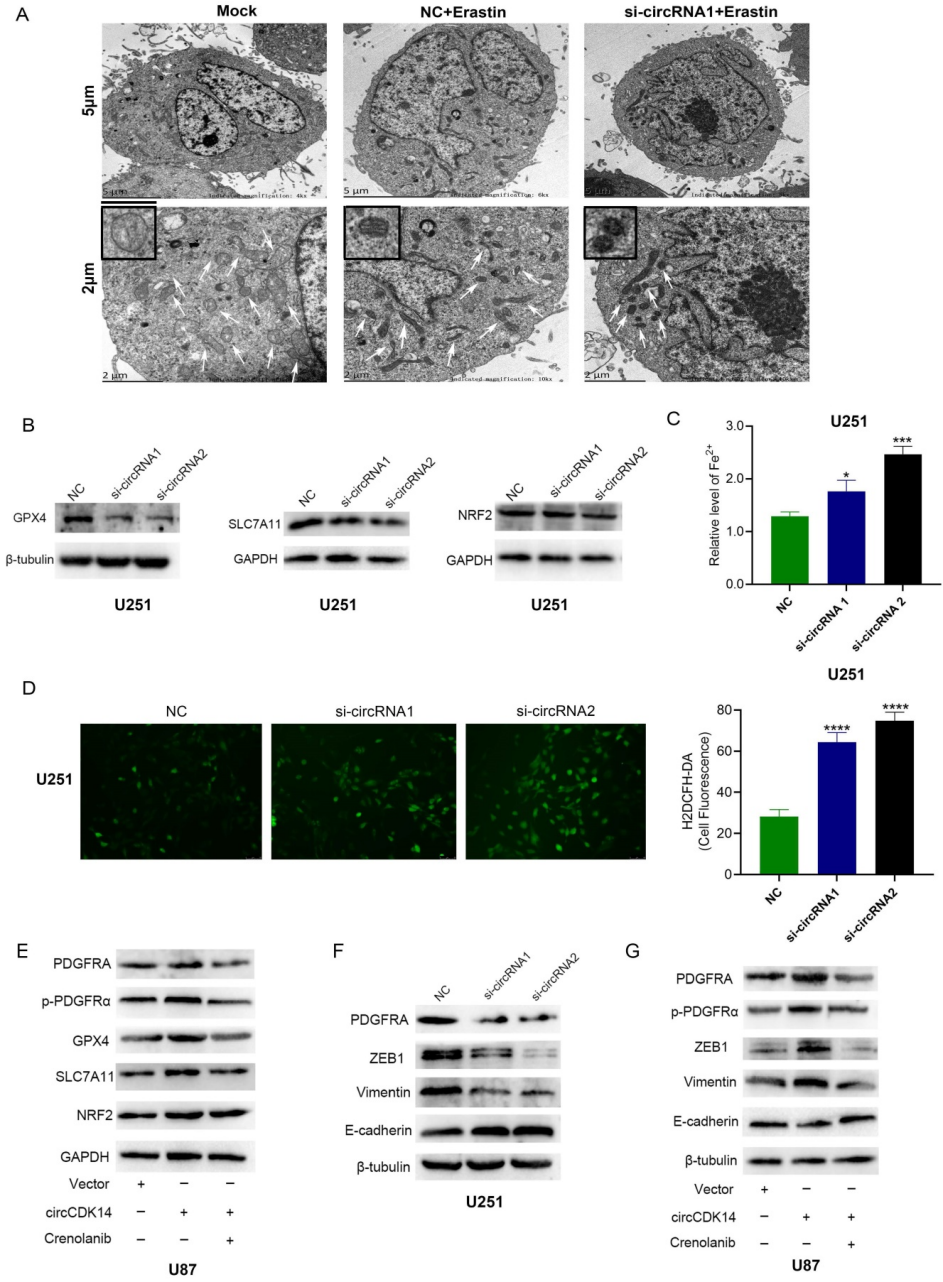
** circCDK14 inhibits glioma cells' sensitivity to Fp. (A)** Transmission electron microscope was used to observe mitochondria in U251 cells with different treatments. Scale bar: 5 µm and 2 µm. In the lower set of figures, arrows point to the mitochondria. The small box in the upper left corner shows the enlarged mitochondria. Mock, control group; NC+Erastin, U251 cells transfected by negative control sequence following 10 µM erastin treatment for 24 h; si-circRNA1+Erastin, U251 cells transfected by circCDK14 interference sequence1 following 10 µM erastin treatment for 24 h. **(B)** GPX4, SLC7A11 and NRF2 protein expressions in U251 cells or circCDK14 knockdown U251 cells were detected by western blot. **(C)** Fe^2+^ level was measured in U251 cells or circCDK14 knockdown U251 cells treatment with 10 µM erastin for 24 h by spectrophotometric method. **(D)** ROS levels were detected in U251 cells or circCDK14 knockdown U251 cells treated with 10 µM erastin for 24 h by H2DCFH-DA fluorescent probe assay. **(E)** The protein levels of PDGFRA, p-PDGFRα, GPX4, SLC7A11 and NRF2 in U87 cells with different treatments, as evidenced by Western blot. Crenolanib, PDGFRα signaling inhibitor. **(F)** The protein levels of p-PDGFRA, ZEB1, vimentin and E-cadherin in U251 cells or circCDK14 knockdown U251 cells, as evidenced by western blot. **(G)** PDGFRA, p-PDGFRα, ZEB1, vimentin and E-cadherin protein levels in U87 cells with different treatments were detected by Western blot. Crenolanib, PDGFRα signaling inhibitor. **(H)** Schematic illustration showing the biological function and mechanism of circCDK14 in glioma. All experiments repeated three times and data averaged and expressed as mean±SD. *p< 0.05, ***p< 0.001, ****p< 0.0001.

To elucidate circCDK14 role in Fp, mitochondria morphology of glioma cells was observed by transmission electron microscopy. Among U251 cells with different treatments, contracting mitochondria, reducing cristaes and increasing membrane density were most obvious in circCDK14 knockdown U251 cells treatment with erastin (Figure 7A). Then Fp related proteins including SLC7A11, GPX4 and NRF2 were detected in U251 cells knockdown circCDK14 with siRNA1 and siRNA2. The results showed that the protein levels of SLC7A11 and GPX4 decreased significantly after depletion of circCDK14, while the expression of NRF2 showed no significant change (Figure 7B). Since massive amounts of lipid ROS and iron are major stimulators of Fp, we measured the Fe^2+^ and lipid ROS levels in U251 cells after 10μM erastin treatment. Based on our data, the Fe^2+^ and ROS levels were markedly elevated in circCDK14 knockdown U251 cells (Figure 7C-D). Together, these data demonstrate that circCDK14 reduced glioma cells' sensitivity to erastin-induced Fp.

Our previous bioinformatic analysis didn't show that SLC7A11 and GPX4 were included in the direct target genes of circCDK14. CircCDK14 influenced the protein expression of SLC7A11 and GPX4 probably depended on PDGFRA. To prove it, PDGFRA, p-PDGFRα, GPX4, SLC7A11 and NRF2 expression were detected by Western blot in glioma cells treated with or without the PDGFRα signaling inhibitor Crenolanib. The results indicated that the expression of PDGFRA, p-PDGFRα, GPX4 and SLC7A11 were significantly increased in circCDK14 overexpression U87 cells. While the expression of PDGFRA, p-PDGFRα, GPX4 and SLC7A11 could be counteracted by Crenolanib (Figure 7E). These data indicate that circCDK14 may reduce glioma cells' sensitivity to Fp by regulating the expression of PDGFRA and PDGFRα signaling.

We also found that epithelial related proteins ZEB1 and Vimentin expression significantly decreased while mesenchymal related protein E-cadherin expression increased in U251 cells with knockdown of circCDK14 (Figure 7F). Meanwhile, overexpression of circCDK14 promoted the expression of PDGFRA, p-PDGFRα, ZEB1 and Vimentin, and inhibited E-cadherin levels. PDGFRA, p-PDGFRα, ZEB1, Vimentin and E-cadherin expression also could be counteracted by Crenolanib (Figure 7G). These results imply that circCDK14 promotes EMT of glioma cells via regulating PDGFRA expression.

### CircCDK14 promotes tumor formation of glioma *in vivo*

**Figure 8:**
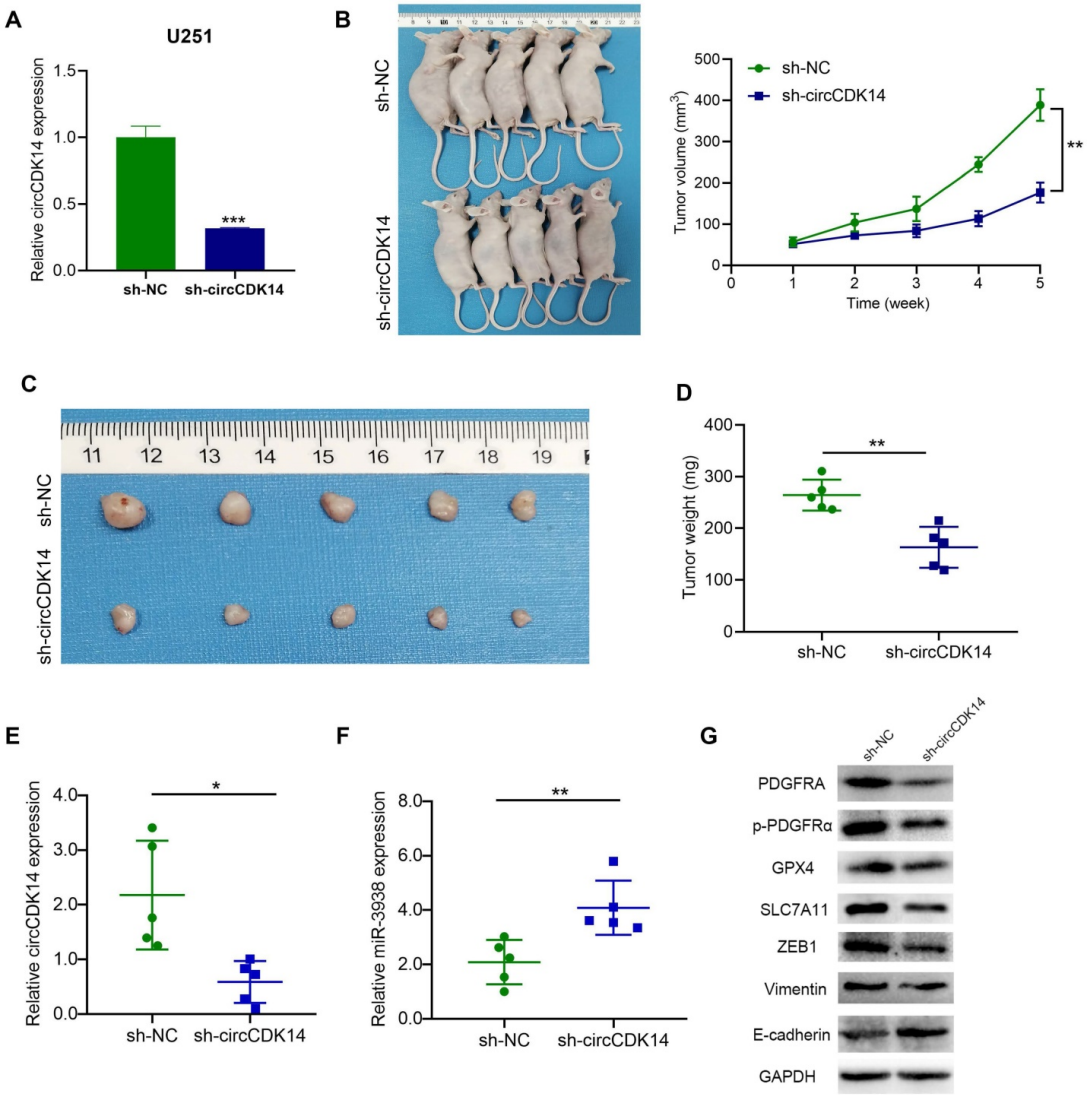
** CircCDK14 promotes the growth of gliomas *in vivo*. (A)** qRT‐PCR analyses for the expression of circCDK14 after stable transfection sh-circCDK14 and sh-NC in U251 cells. **(B)** The volume of subcutaneous tumor was measured every seven days. Formula: V(mm^3^) = length× width^2^/2. **(C)** Photographs of subcutaneous glioma tumors in nude mice (n=5 each group). **(D)** Tumor weight was markedly reduced in sh-circCDK14 group, relative to controls. **(E and F)** qRT-PCR detects the circCDK14 and miR-3938 levels in nude mice tumors from different groups. **(G)** Western blotting demonstrated the protein levels of PDGFRA, p-PDGFRα, ZEB1, Vimentin, E-cadherin, SLC7A11 and GPX4 in nude mice tumors from different groups. All experiments repeated three times and data averaged and expressed as mean±SD. *p< 0.05; ***p< 0.001; ****p< 0.0001.

To further investigate whether circCDK14 contribute to tumor progression* in vivo*, U251 cells stably transfected with sh-circCDK14 or sh-NC (Figure 8A). Nude mice were subcutaneously implanted with stably transfected U251 cells and tumor development was left uninterrupted for 35 days. Based on our analysis, circCDK14 silencing substantially reduced the xenograft tumor growth rate, compared to the control group (Figure 8B). Decreased tumor sizes and weights were also apparent in sh-circCDK14 mice, compared to sh-NC mice at the same endpoint (Figure 8C,8D). Next, circCDK14 and miR-3938 expression of the tumor were detected by qRT-PCR and found that the expression level of circCDK14 was higher in the sh-NC group, while the expression level of miR-3938 was higher in the sh-circCDK14 group (Figure 8E,8F). Meanwhile, the protein levels of PDGFRA, p-PDGFRα, ZEB1, Vimentin, E-cadherin, SLC7A11 and GPX4 were assessed by Western Blot. The results also demonstrated that expression of PDGFRA, p-PDGFRα, ZEB1, Vimentin, SLC7A11 and GPX4 were lower in circCDK14-knockdown tumors than that in control tumors, and E-cadherin expression was negative associated with circCDK14 expression (Figure 8G).

## Discussion

Glioma is a highly prevalent intracranial tumor with resistance to multiple therapies and poor OS. Unfortunately, glioma etiology remains a mystery. It may be the result of an intricate network of numerous genes, signaling pathways and molecular components. None-coding RNAs like miRNA, lncRNA and circRNA constitute a considerable portion of the human transcriptome and are known to play a role in glioma development and progression. Emerging evidences point to circRNAs prevalence in neuronal tissues, likely due to the abundance of circularization-specific genes. Hence, it is possible that abherrant circRNAs may lead to diseases of the neuronal system like glioma 31. However, the underlying mechanisms are yet to be determined.

Herein, we found that circCDK14 was upregulated in glioma by RNA-seq and qRT- PCR. CircCDK14 is derived from the known protein-coding gene CDK14. As the host gene of circCDK14, CDK14 acts as a cyclin-dependent kinase that regulates cell cycle progression and cell proliferation. CDK14 involves in tumorigenesis of various cancers including glioma 30, 32, 33. Studies shows that the interaction between CDK14 and cyclin Y can promote noncanonical Wnt signaling in human hepatocellular carcinoma 34. CDK14 silencing strongly suppressed pancreatic cancer cell proliferation and invasion, as well as EMT progression via inhibition of the PI3K/Akt signaling pathway 35. However, the circRNAs derived from CDK14 as well as their function in cancer progression are still poor understood. Here, the gain-and-loss function assays showed that circCDK14 is involved in the progression of glioma via modulating the proliferation, migration and invasion of cancerous cells. Through analysis of more clinical samples, we found that an abundance of cicCDK14 was strongly associated with poor OS and tumor formation experiments *in vivo* also strongly hints towards its functions in glioma progression.

CircRNAs are most frequently reported to contain miRNA docking sites and may sequester the miRNA-mediated inhibition of target genes. Herein, we demonstrated that circCDK14 primarily a cytoplasmic RNA in glioma cells. RNA-pull down, FISH and luciferase reporter assays further confirmed the authentic interaction between circCDK14 and miR-3938. We demonstrated that miR-3938 not only has a docking site for circCDK14 in glioma cells but also demonstrated to reverse circCDK14 functions in glioma. Moreover, our data indicates that circCDK14 modulates glioma activity through PDGFRA regulation via miR-3938. PDGFRA is a classical proto-oncogene that encode receptor tyrosine kinases responding to platelet-derived growth factor. It is a transmembrane receptor carrying 5 immunoglobulin-like repeats in its extracellular domain and a tyrosine kinase in its intracellular domain. Upon activation with a ligand, the receptor initiates crucial downstream pathways, namely MAP kinase, PI3K/AKT, JAK/STAT, and PLC-PKC that induce oncogenesis 36. Like EGFR, PDGFRA was reported to be highly expressed, amplified, mutated, or truncated in gliomas 37-40. Our findings indicated that circCDK14 behaves like a ceRNA to accelerate glioma progression via the miR-3938/PDGFRA axis.

Fp is programmed cell death whereby iron-based lipid hydroperoxides accumulating to lethal levels 41. Multiple human diseases including cancer are associated with abberant Fp functions. The metabolite-based Fp involve GPX4 (Glutathione peroxidase 4) and system Xc^-^, which imports cysteine and exports glutamate. GPX4 is the one unique enzyme in mammalian cells that can remove lipid ROS with reduced glutathione (GSH) as a substrate. Overexpression of GPX4 in cells confers resistance to Fp, while knockdown of GPX4 promotes Fp 42. On the other hand, compounds that suppress the cystine-glutamate antiporter (and ultimately reduce GSH levels) or downregulate GPX4 activity strongly activate Fp. Apart from system Xc^-^ and GPX4, other genes are also known to regulate cell responsiveness to Fp such as transcription factor nuclear factor erythroid 2-related factor 2 (NRF2). NRF2 is a crucial modulator of cellular antioxidant response, and controls transcription of genes involved in the anti-oxidative and anti-electrophilic stresses as well as Fp 43, 44. To further examine the correlation between PDGFRA and Fp, we performed functional enrichment and Fp potential index model analysis. Based on our data, PDGFRA expression was negatively correlated with Fp. Because circCDK14 could regulate PDGFRA expression via miR-3938, the roles of circCDK14 in Fp also were explored. After knockdown of circCDK14 in glioma cells, protein levels of SLC7A11 and GPX4 decreased significantly and Fp became more sensitivity. Those data indicate that circCDK14 may reduce glioma cells' sensitivity to Fp by regulating the expression of PDGFRA.

All in all, our data demonstrated a previously unknown oncogenic role of circCDK14 in glioma pathogenesis. However, we encountered certain limitations. For example, whether circCDK14 can be accurately detected in body fluids and shows potential value as tumor markers for glioma diagnosis need further investigation. We will continue to explore the detail mechanisms of circCDK14 reducing glioma cells' sensitivity to Fp and provide a new insight as therapeutic strategies for glioma treatment.

In conclusion, we demonstrated that circCDK14 is overexpressed in glioma tissues and cell lines, and the elevated expression of circCDK14 is strongly associated with the poor OS of glioma patients. Examining its underlying mechanism, we showed that circCDK14 accelerates glioma progression by sequestering miR-3938 and ultimately regulating PDGFRA, a well-known glioma oncogene. Moreover, circCDK14 reduced glioma cells' sensitivity to Fp by regulating the expression of PDGFRA. We revealed for the first time the biological activity of circCDK14 in glioma, suggesting that the circCDK14/PDGFRA axis is an excellent candidate for targeted anti-glioma therapy.

## Supplementary Material

Supplementary figures and tables.
