# Supplementary material for: CircCDK14 Promotes Tumor Progression and Resists Ferroptosis in Glioma by Regulating PDGFRA
Source: Int J Biol Sci. 2022 Jan 1;18(2):841–57. doi: 10.7150/ijbs.66114 (PMC8741855; doi:10.7150/ijbs.66114)
Supplement: Supplementary file 1 — Supplementary figures and tables. [file ijbsv18p0841s1.pdf]

Supplementary Information

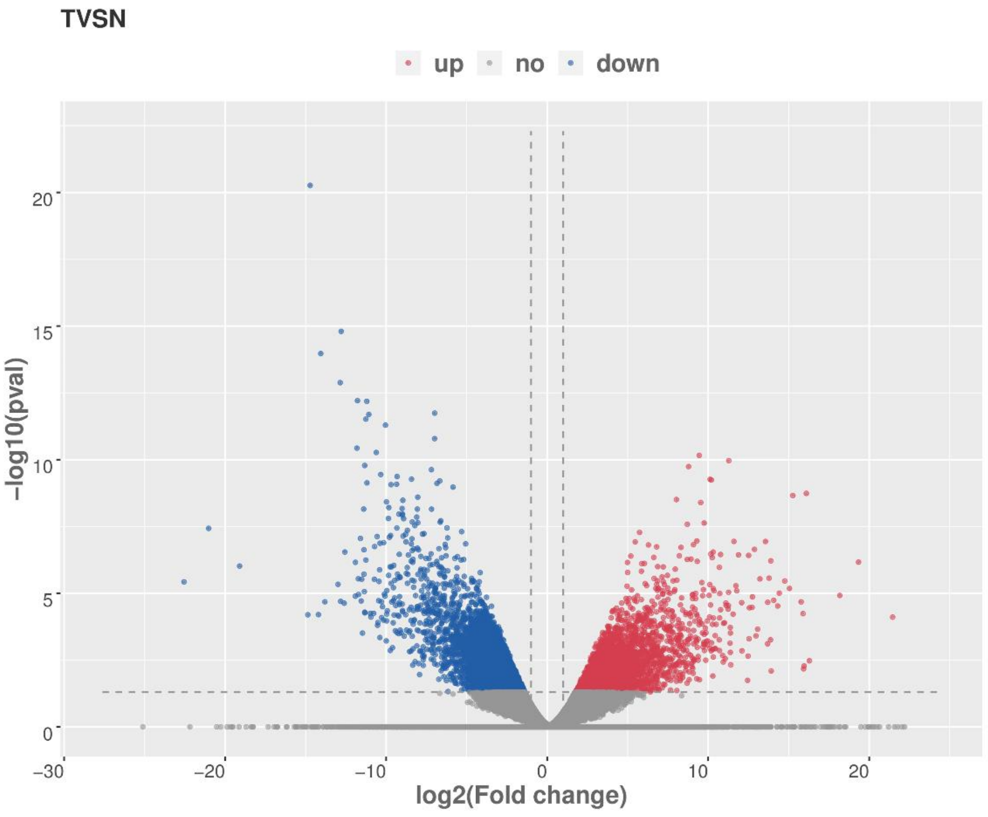

Supplementary Figure 1. Volcano plot describing the profile of circRNAs expression in glioma tissue and normal brain tissue.

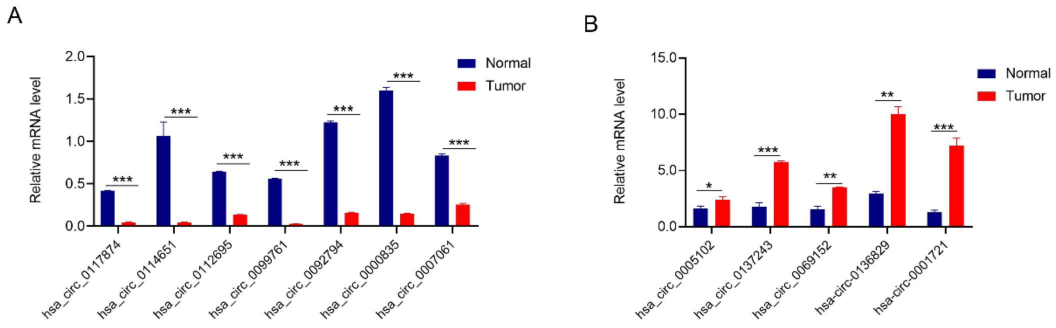

Supplementary Figure 2. The results of RNA-Seq were confirmed by qPCR in 9 sequencing samples.

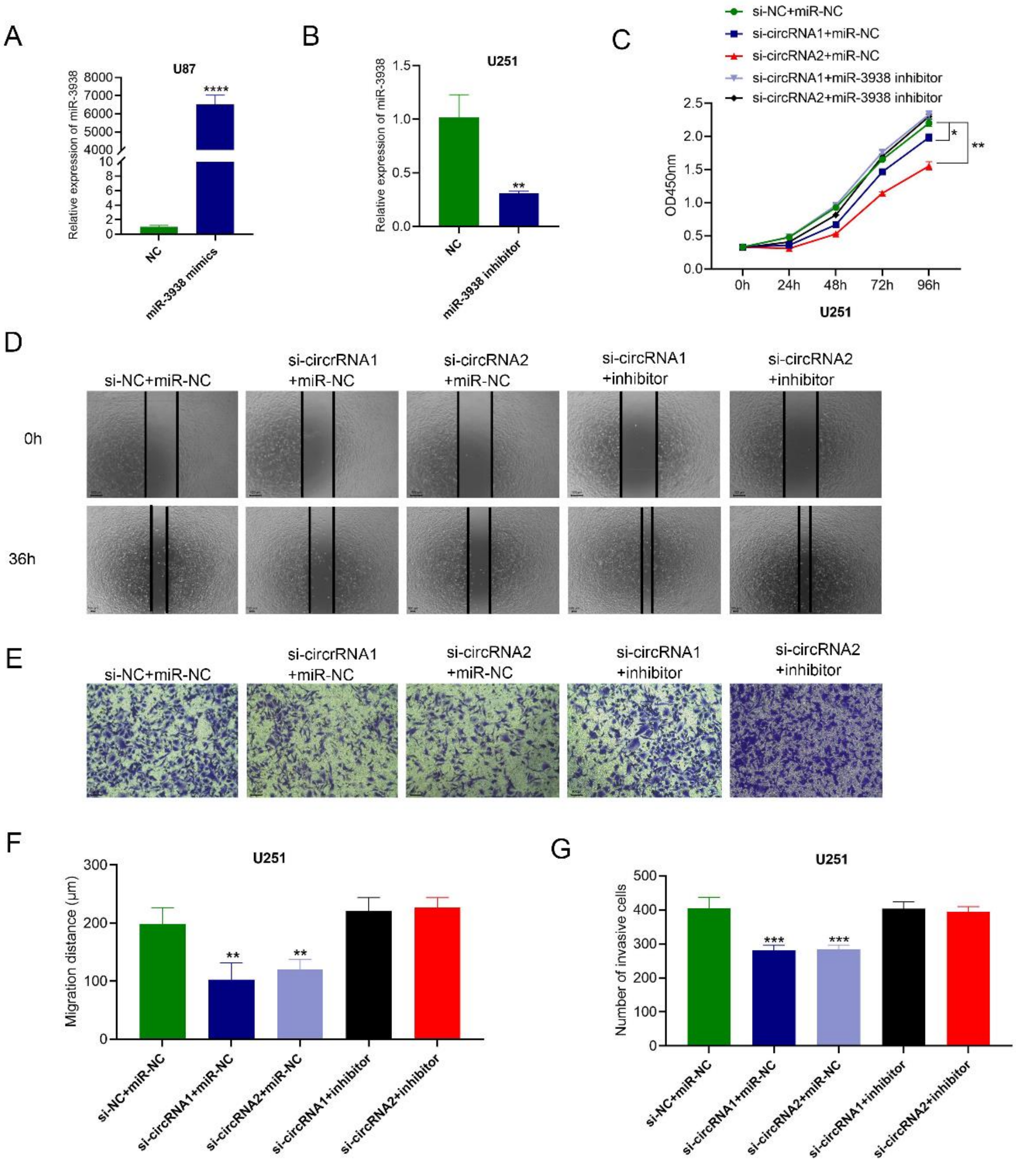

Supplementary Figure 3. The inhibition of miR-3938 expression could counteract the abilities of proliferation, migration and invasion in circCDK14 knockdown glioma cells.

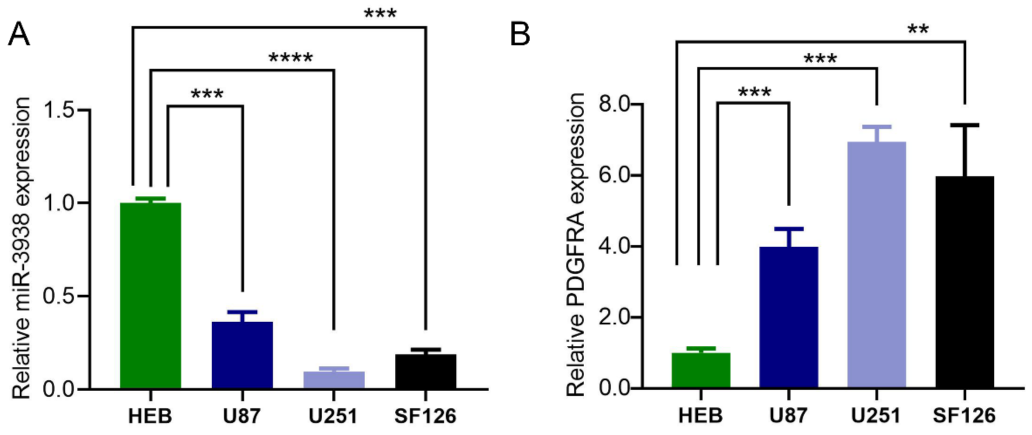

Supplementary Figure 4. The level of miR-3938 and PDGFRA in glioma cells.

Table S1 Primer sequences

Genes	Primer	Sequence (5’-3’)
circCDK14 (divergence)	Forward primer	CTCCTCCACTGGCAAAGAG
	Reverse primer	GTGTAGACATCTTTGTGACACATATCGAG
circCDK14 (convergence)	Forward primer	TGATCAAACCCCTGGACACA
	Reverse primer	GCCAGTGGAGGAGGTCTTAA
GAPDH (convergence)	Forward primer	GTCTCCTCTGACTTCAACAGCG
	Reverse primer	ACCACCCTGTTGCTGTAGCCAA
GAPDH (divergence)	Forward primer	TGTTCTGTTTCGTGTGTGAGG
	Reverse primer	TGTGAGAAGCCTCTGTTCGT
CDK14	Forward primer	TGTCTTACATCCACCAGCGT
	Reverse primer	TGTGGCTAGGGACGGATTTT
has_circ_0117874	Forward primer	GAAGGACCATGCTCAGAAGC
	Reverse primer	ATTAACTCTTCCATTGCAGC
has_circ_0114651	Forward primer	TCACTGCAAAGACCACACAG
	Reverse primer	GGCGTTCCAGTTTCATGCG
has_circ_0112695	Forward primer	GCCATTCAAACTGCTATCCG
	Reverse primer	GTAACAATCCTGACTTGGTAGCT
has_circ_0099761	Forward primer	AGATCTGGGACTTAGCAGGC
	Reverse primer	GCAAGCACAAAACCACAGTCTA
has_circ_0092794	Forward primer	TTTCCTGGCCTATTAAAATACAGATG
	Reverse primer	TTCGACTGTTCTGGGTTTG
has_circ_0000835	Forward primer	ATCCAAGTGGCAATAGGCAT
	Reverse primer	GCACCTGCAAAGATTCCTC
has_circ_0007061	Forward primer	AATGGTGACAGATCCAGGCA
	Reverse primer	GATTCCAGCAGCCTCCTG
has_circ_0005102	Forward primer	GGGGCAGGCAATAAATGGAGC
	Reverse primer	AGACTGGACGTTCTACAGGC
has_circ_0137243	Forward primer	TTTGCGGAGGAAGACTATACC
	Reverse primer	CTTGCCCCTCCCAGTTT
has_circ_0069152	Forward primer	GAAGGACCATGCTCAGAAGC
	Reverse primer	ATTAACTCTTCCATTGCAGC
has_circ_0136829	Forward primer	CAGAAGGCCCAGTGGTAGAA
	Reverse primer	GCTTCTGAGTCAGGTTTCTTATTAAAG
miR-3938	Stem-loop primer	GTCGTATCCAGTGCAGGGTCCGAGGTATTCGCACTGGATACGACCCGGGT
miR-3938	Forward primer	GCGCGAATTCCCTTGTAGATA
	Reverse primer	CTCGCTTCGGCAGCACA
U6	Forward primer	AACGCTTCACGAATTTGCGT
	Reverse primer	CTCGCTTCGGCAGCACA
PDGFRA	Forward primer	ATGGATTAAGCCGGTCCCAA
	Reverse primer	TAAATGGGGCCTGACTTGGT

Table S2 Correlation between circCDK14, miR-3938 expression and clinicopathological features of patients.

Characteristics	Case	**CircCDK14 expression**		P value	**miR-3938 expression**		P value
		**High**	**low**		**High**	**low**	
All cases	76	37	39		38	38	
Age(years)				0.643			0.486
>50	34	17	15		14	18	
<=50	42	20	24		24	20	
Gender				0.359			0.358
Male	38	21	17		21	16	
Female	38	16	22		17	22	
Grade				0.027			0.002
I-II	30	9	23		22	8	
III-IV	46	28	16		16	30	
